# Effect of Media with Different Glycerol Concentrations on Sheep Red Blood Cells’ Viability In Vitro

**DOI:** 10.3390/ani11061592

**Published:** 2021-05-28

**Authors:** Valeria Pasciu, Francesca D. Sotgiu, Cristian Porcu, Fiammetta Berlinguer

**Affiliations:** Department of Veterinary Medicine, University of Sassari, 07100 Sassari, Italy; fran.sotgiu@gmail.com (F.D.S.); cporcu@uniss.it (C.P.); berling@uniss.it (F.B.)

**Keywords:** glycerol, sheep erythrocytes, osmotic stress, oxidative stress, ATP, Ca^2+^, hemolysis

## Abstract

**Simple Summary:**

Glycerol is widely used as a feed supplement in ruminant nutrition. However, its administration at moderate and high doses results in an increase in plasma osmolality and in an alteration of red blood cell (RBC) indices. The present study aimed at further elucidating the effect of glycerol on RBCs’ functionality by evaluating the effect of different glycerol concentrations on RBC homeostasis under in vitro conditions. Obtained results showed that glycerol permeates the RBC membrane and leads to hemolysis when glycerol concentration exceeds 200 mg/dL. However, RBCs’ antioxidant defenses appear to protect cell membranes without causing an increase of oxidative stress markers. Moreover, no alteration in RBCs’ intracellular Ca^2+^ ion concentrations and metabolic activity were found. In conclusion, glycerol-based nutritional treatment should be designed in sheep to avoid exceeding 200 mg/dL glycerol circulating concentration to prevent RBC osmotic stress.

**Abstract:**

The use of high doses of glycerol as a livestock feed supplement is followed by a rapid increase in plasma concentrations and consequently in plasma osmolality. Moreover, glycerol is a highly diffusible molecule that can readily permeate the red blood cell (RBC) membrane following a concentration gradient. A rise in glycerol plasma concentrations can thus alter RBC homeostasis. The present study aimed at investigating both glycerol osmotic effects on sheep RBCs and their oxidative response under in vitro conditions. Sheep blood samples were suspended in media supplemented with increasing glycerol concentrations (0, 25, 50, 100, 150, 200, 250, 300, 350, 400 mg/dL), which reflected those found in vivo in previous studies, and incubated at 37 °C for 4h. Thereafter, osmolality and hemolysis were determined in spent media, while cell extracts were used to assay intracellular concentration of glycerol, ATP, Ca^2+^ ions, oxidative stress markers and reactive oxygen species (ROS).The study confirmed that glycerol intracellular concentrations are directly related with its concentration in the incubation media, as well as hemolysis (*p* < 0.001) which increased significantly at glycerol concentrations higher form 200 mg/dL. ROS intracellular level increased at all glycerol concentration tested (*p* < 0.01) and total thiols decreased at the highest concentrations. However, RBCs proved to be able to cope by activating their antioxidant defense system. Superoxide dismutase activity indeed increased at the highest glycerol concentrations (*p* < 0.001), while total antioxidant capacity and malonyldialdehyde, a typical product of lipid peroxidation by ROS, did not show significant changes. Moreover, no alterations in intracellular Ca^2+^ ions and ATP concentrations were found. In conclusion, glycerol-induced hemolysis can be related to the induced osmotic stress. In sheep, nutritional treatments should be designed to avoid reaching glycerol circulating concentrations higher than 200 mg/dL.

## 1. Introduction

Glycerol is widely used as a feed supplement in small ruminant nutrition, both as replacer of corn grain in the diet [1,2,3,4,5] and to increase productive performance [6,7,8,9,10,11,12]. Glycerol is a hyperosmotic agent, lipid soluble, and it can pass cell membrane by simple diffusion, following a concentration gradient [13,14]. In previous studies [15,16], we reported that oral administration of glycerol-based formulation in ewes causes a significant increase in circulating glycerol concentration (from 14.19 to 289.8 mg/dL) and in plasma osmolality (from 0.316 to 0.378 osmol/kg). These changes were associated with significant alteration in red blood cell (RBC) indices with a dose-related effect, which included shape alterations (increase in RBCs volume and distribution width) and a dilution in the amount of hemoglobin per volume unit [16]. These alterations were found 2 h after the oral administration of glucogenic formulations when glycerol concentrations in the bloodstream were higher than ≈ 200 mg/dL. Similar effects were described in in vitro human glycerolized RBCs and were associated with an impaired functionality and reduced cell deformability, which in turn can affect the cells’ capability to enter and transit the capillaries and to exchange gases, nutrients, and cell waste [17,18].

The previously reported changes in RBC volume homeostasis consequent to the increase in glycerol concentration in the bloodstream are driven by glycerol entering the cells and by the consequent water drawing [19]. Changes in RBCs’ volume homeostasis can affect their membrane integrity, leading to impaired functionality and increased eryptosis [20,21]. Previous studies indeed reported that glycerol is able to induce hemolysis of mammalian RBCs, likely consequent to the induced osmotic stress [22,23].

The above-mentioned alterations in RBC volume homeostasis may also result in oxidative stress [24]. In various cell types, ROS production is increased following hypotonic exposure [25,26]. Oxidative stress is caused by increasing productions or decreasing elimination of reactive oxygen species (ROS) in the cells [24], and, therefore, it can also be described as an imbalance between the prooxidants and antioxidants in the cells. Malonyldialdehyde (MDA), a typical product of lipid peroxidation by ROS, can crosslink phospholipids and proteins and oxidize protein sulfhydryl groups, thus damaging the cell membrane and causing hemolysis [27]. Moreover, both oxidative stress and hyperosmolarity can causes eryptosis by determining an increase in Ca^2+^ ions in the cytosol by the activation Ca^2+^ penetration through non-selective cation channels [28]. Normally cells have efficient antioxidant defense systems to face oxidative stress. Reduced glutathione (GSH) is the primary RBCs endogenous antioxidant and, given its ability to detoxify hydrogen peroxide and others electrophilic compounds, it is in the first line of defense against ROS. GSH have been reported to play a crucial role in maintenance in reduced state of hemoglobin -SH groups and in other enzymes [29]. However, the cellular redox defense system can also be altered by hyperosmotic conditions though a depletion in intracellular glutathione (GSH) availability [30]. Other antioxidant enzymes as superoxide dismutase (SOD) also provide primary protection from oxidative stress. SOD protects cells from toxic oxygen metabolites by converting superoxide radical into molecular oxygen and hydrogen peroxide. Superoxide radical is one of the more representative ROS in cells, and consequently SOD plays a key role on antioxidant defense [31]. Furthermore, the cells have a parallel non-enzymatic antioxidant system, and the Trolox equivalent antioxidant capacity (TEAC) is used to evaluate its capacity to face oxidative stress [32].

Starting from these premises, we hypothesized that elevated plasma glycerol can alter RBC homeostasis directly through changes in RBC volume and indirectly by leading to oxidative stress. The present study was thus designed to elucidate these direct and indirect consequences of RBCs’ exposure to glycerol. RBCs were incubated in vitro with increasing glycerol concentrations, in the range of those found in in vivo studies after glycerol oral administration in ewes [15,16]. Cell lysis was measured and oxidative status was evaluated by assaying ROS intracellular concentration, SOD activity, total thiols, TEAC, and MDA in cell extracts. Moreover, to assess whether the alterations found could trigger eryptosis in vivo and alter cell metabolisms, intracellular concentration of Ca^2+^ ions and ATP were determined. Furthermore, to evaluate the possible effect of changes in cell volume on ROS production, sheep RBCs were exposed to hyper-osmotic and hypo-osmotic solutions and their intracellular ROS concentration was determined. Results obtained aimed at elucidating the possible side effects of glycerol administrations in ruminants on RBC viability.

## 2. Materials and Methods

### 2.1. Reagents and Animals

Unless otherwise specified, all chemical and reagents were purchased from Sigma. All procedures involving animals in this study were approved by the Local Animal Care and Use Committee (authorization code: 2899 of 17/01/2018). Ewes were confined outdoors with access to a sheltered area, at the experimental facilities of the Department of Veterinary Medicine at the University of Sassari, Italy, IT (40°43′40.33″ N, 8°33′1.33″ E). Blood samples from four ewes were collected at fasting (07:00 a.m.) from the jugular vein using 9 mL vacuum collection tubes containing EDTA K2 (Vacutainer Systems Europe; Becton Dickinson, Meylan Cedex, France). Mean erythrocyte concentration was 12 × 10^9^/mL. Blood was then diluted to 0.12 × 10^9^ cells/mL with Hanks’ Balanced Salt Solution (HBSS; Sigma code H6648). Whole blood was portioned in 10 samples and then diluted at the concentration of 0.10 × 10^6^ RBCs/µl (0.10 × 10^9^/mL) with HBSS supplemented with increasing glycerol concentrations (0, 25, 50, 100, 150, 200, 250, 300, 350, 400 mg/dL). Samples were then incubated at 37 °C during 4 h. Glycerol stock solutions were prepared at 90% using H_2_O and vegetal Glycerol at 99.5% by Farmalabor (Canosa di Puglia, Provincia di Barletta-Andria-Trani, Italy; cod 018916). Following the incubation period, blood samples were gently centrifuged (100 g for 3 min), and blood cells were separated from supernatant. Blood cell were used for the assay of intracellular glycerol, SOD, TEAC, total thiols, MDA, ATP, and CA^2+^ ions while supernatant was used for hemolysis. A separate set of samples was diluted at 7 × 10^6^ RBCs/mL with glycerol and HBSS and incubated at 37 °C during 4 h for ROS assay. All analyses for each sample (1 sample/ewe) were repeated in 4 replicates.

### 2.2. Intracellular Glycerol Assay

Glycerol assay was performed using Cell Biolabs’ Free Glycerol Assay Colorimetric Kit (DBA Italia, Segrate, Milan, Italy; STA-398). Triton X100 extract RBC was used for measuring intracellular glycerol by a coupled enzymatic reaction system. The glycerol was phosphorylated and oxidized, producing hydrogen peroxide which reacts with the kit’s Colorimetric Probe with maximum absorbance at 570 nm [16]. The glycerol concentrations were calculated using a standard curve with seven different points of standard glycerol concentrations from 0.06 to 3.68 mg/dL and expressed in µg/10^9^ erythrocytes.

### 2.3. Haemolysis

The absorbance of the hemoglobin in the supernatant was assessed at 405 nm. The absorbance of the supernatant of sheep RBC lysed in H_2_O was taken as 100% hemolysis [33].

### 2.4. Osmolality Measurement

Dilution media osmolality measurement (Osm/kg) was performed using a freezing point osmometer (Osmomat 030, Gonotec, Berlin, Germany).

### 2.5. ROS Assay

Blood samples diluted at the concentration of 7 × 10^6^ RBCs/mL were added with the 2′,7′-dichlorodihydrofluorescein diacetate (H_2_DCF-DA) probe at the final concentration of 3 µM. Within the cell, the esterases cleave the acetate groups on H_2_DCF-DA, thus trapping the reduced form of the probe 2′,7′-dichlorodihydrofluorescein (H_2_DCF). Intracellular ROS oxidize H_2_DCF, yielding the fluorescent product, DCF. Fluorescence was measured using FLUOstar Omega microplate reader (BMG LABTECH). Excitation and emission wavelengths used for fluorescence quantification were 485 and 535 nm, respectively. All fluorescence measurements (relative fluorescence unit—RFU) were corrected for background fluorescence. Data were expressed as means ± SE [34,35].

### 2.6. SOD, TEAC, Total Thiols, and MDA Assays

Blood cells were treated with HBSS containing TritonX100 to 0.1% to obtain the cellar extract samples for SOD, TEAC, MDA, and total thiols assays. These assays were performed as reported in previous works [36,37]. All data were normalized for total cells and were assayed using an automatic cell counter instrument (Hematology analyzer Alcyon Mindray BC-5000, Shenzhen, China).

#### 2.6.1. SOD Assay

SOD activity was assayed in cellular extracts and was measured enzymatically (at 470 nm) as a decrease of the XTT (3′-(1- [(Phenylamino) -carbonyl] -3,4-tetrazolium) –bis (4-methoxy-6-nitro) benzenesulphonic acid hydrate) reduction by superoxide anion generated by xanthine oxidase [38].

The values of SOD in the samples were calculated using a standard curve (0.065–0.8 U/mL) and expressed in U/10^9^ erythrocytes. The number of erythrocytes was considered, taking the % of hemolysis for each group, and one enzyme unit (IU) was defined as the amount of SOD capable of transforming 1.0 mmole/min of O_2•−_.

#### 2.6.2. Total Thiols

**Figure 1:**
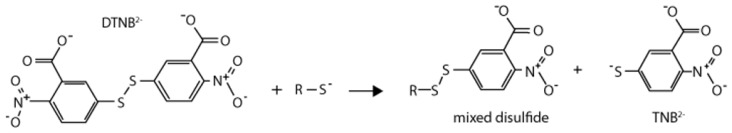
Ellman’s reaction.

Total Thiols was assayed using the Ellman’s Reagent 5,5-dithio-bis-(2-nitrobenzoic acid) (DTNB) solved in PBS. Thiols react with this compound (Figure 1), cleaving the disulfide bond to give 2-nitro-5-thiobenzoate (TNB−), which ionizes to the TNB^2−^ dianion in water at neutral and alkaline pH. This TNB^2−^ ion has a yellow color.

The TNB2—was quantified in a spectrophotometer by measuring the absorbance of visible light at 412 nm, using an extinction coefficient of 14,150 M^−1^ cm^−1^ for dilute buffer solutions [39]. The values of Thiols in the samples were expressed in nmol/10^9^ erythrocytes.

#### 2.6.3. TEAC Assay

TEAC was determined using the method described by Re et al. and modified by Lewinska et al. [40]. Briefly, a fresh solution was prepared by dissolving 19.5 mg 2,20-azinobis (3- ethylbenzthiazoline -6-sulphonic acid [ABTS]) and 3.3 mg potassium persulphate in 7 mL of 0.1 mol/L phosphate buffer, pH 7.4. This solution was stored in the dark for 12 h for completion of the reaction. ABTS solution was diluted (usually approximately 1:80) in 0.1 mol/L phosphate buffer, pH 7.4, to give an absorbance reading at 734 nm of 1.0 and mixed thoroughly. The antioxidant capacity was expressed as TEAC, that is, the concentration of trolox producing the same effect as the sample studied. The values of TEAC in the samples were calculated using a standard curve (5–20 mM trolox in a total volume of 550 mL) and were expressed as µmoles of trolox equivalent/Cell extract (µmolTEAC/10^9^ erythrocytes).

#### 2.6.4. MDA Assay

MDA, one of the several low-molecular-weight end-products of lipid peroxidation, was evaluated by the TBARS assay using thiobarbituric acid and a spectrophotometric method according to the TBA test described by Spanier and Traylor [41], with some modifications. The values of MDA in the samples were calculated using a standard curve (2–100 µM) and expressed in nmol/10^9^ erythrocytes.

### 2.7. Ca^2+^ Ions Assay

The determination of intracellular Ca^2+^ ions concentration was performed using the Ca^2+^ ions Assay Kit (Hagen Diagnostica cod. 001-0037). HBSS containing CaCl_2_ 1mM and MgCl_2_ 0.5 mM (0.285 osmol/Kg) was used for blood dilution. After incubation with different concentrations of glycerol and centrifugation, blood cells were treated with PBS containing TritonX100 0.1% and the cell extract was assayed (at 570nm) with a direct colorimetric assay based on the O-Cresolphtalein (OCPC) method without deproteinization of the sample [42].

Ca^2+^ ions concentrations were expressed in nmol/10^9^ erythrocytes using a Ca^2+^ ions standard of 25 µM and normalized for cell number.

### 2.8. Extraction and Measurement of Intracellular ATP

Determination of intracellular ATP concentration was performed by the enzymatic assay as described by Bergmeyer et al. [43]. Briefly, cells were treated with 0.1 mL of ice-cold 0.6 M perchloric acid added to each Eppendorf tube containing erythrocytes with different glycerol concentrations and kept for 15 min, for the extraction of nucleotides. After, the suspension was centrifuged in an Eppendorf Microfuge (3 min at 6000 g) and the supernatant was neutralized with 15 μL of 3.5 M K_2_CO_3_ [44] and measured spectrophotometrically at 340 nm using NADH-linked enzyme-coupled assays. The glucose 6 phosphate dehydrogenase (G6PD) and hexokinase (HK) were used with glucose, nicotinamide adenine dinucleotide phosphate (NADP+), sample (25 μL), all in TRAP buffer (0.1 M, pH 7.6). ATP was determined from the formation of NADPH. The values of ATP in the samples were calculated using a standard curve (0.055–50 µM) and expressed in nmol/10^9^ erythrocytes.

### 2.9. ROS Production Using Solutes with Different Osmolality without Glycerol

Blood samples were diluted at the concentration of 7 × 10^6^ RBCs/mL in 5 different hyper-osmotic solutions and 6 different hypo-osmotic solutions (Table 1). The hyperosmotic solutions were prepared with the addition of an impermeant solute (starch from Merck, C.I.20470), covering the range of osmolarity induced by glycerol in this work. Hypo-osmotic solutions were prepared by adding purified water to a PBS solution to obtain the osmolarity range reported in Table 1. After dilution, blood samples were incubated at 37 °C during 4 h. At the end of the incubation period, ROS production was determined as above described.

### 2.10. Statistical Analysis

Data are expressed as means ± SE. Results were analyzed by a one-way ANOVA test, used to compare intracellular glycerol, oxidative stress markers, and hemolysis, using Minitab 17 Statistical Software (Minitab, Inc., 2010, State College, PA, USA). As post-hoc test, Tukey’ test was used to highlight differences between groups. Statistical significance was accepted at *p* < 0.05. The number of RBCs used to express the results was corrected taking into consideration the % of hemolysis in each group.

## 3. Results

The osmolality of dilution media supplemented with increasing glycerol concentrations is shown in Table 2. As expected, media osmolality increased proportionally with glycerol concentration. These osmolality values are in the range of those found in vivo (from 0.316 to 0.378 osmol/Kg) [16] in ewe’s plasma after oral administration of glycerol as nutritional supplement.

**Figure 2:**
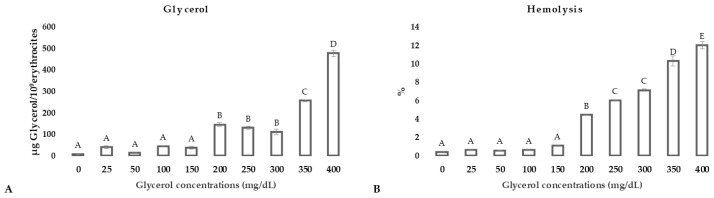
Intracellular glycerol concentrations after RBC incubation in media supplemented with glycerol in concentrations ranging from 0 to 400 mg/dL (**panel A**); Hemolysis (%) after RBC incubation in media supplemented with glycerol in concentrations ranging from 0 to 400 mg/dL (**panel B**). Uppercase letters indicate significant differences between groups (*p* < 0.001; one-way ANOVA–Tukey post hoc test).

Intracellular glycerol concentrations significantly increased after RBC incubation in media containing glycerol at concentrations higher than 200 mg/dL (*p* < 0.01; Figure 2, panel A). At the same concentration range (from 200 to 400 mg/dL), hemolysis significantly increased (*p* < 0.01; Figure 2, panel B).

**Figure 3:**
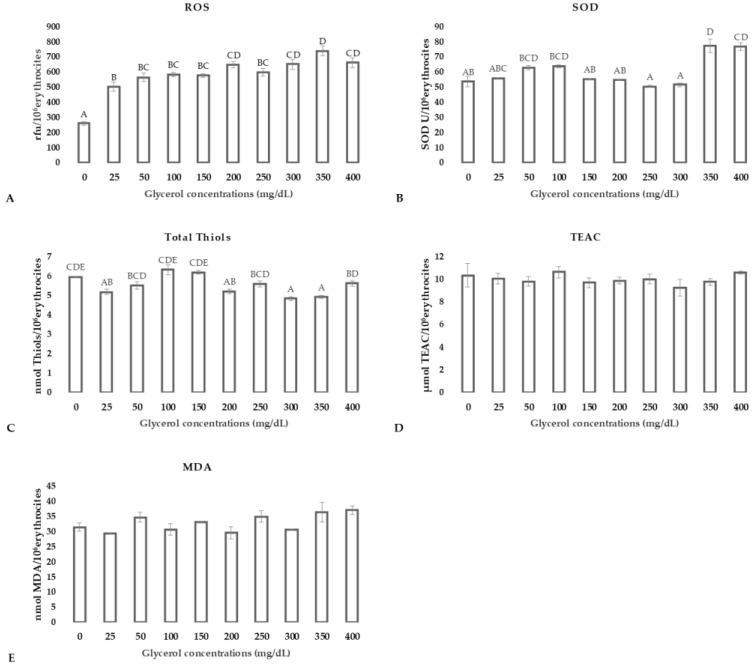
ROS production (**panel A**), SOD activity (**panel B**), total thiol concentrations (**panel C**), total anti-oxidant capacity (TEAC; **panel D**), and MDA (**panel E**) concentration in RBCs after incubation in media supplemented with glycerol in concentrations ranging from 0 to 400 mg/dL. Uppercase letters indicate significant differences between groups: ROS *p* < 0.01; TEAC *p* = 0.764; total thiols *p* < 0.05; SOD *p* < 0.05; MDA *p* = 0.062 (one-way ANOVA–Tukey post hoc test).

ROS production increased significantly in RBC incubated with all glycerol supplemented media compared to the control (*p* < 0.01; Figure 3, panel A). In contrast SOD activity increased significantly only at high glycerol concentrations (350 and 400 mg/dL; *p* < 0.001 Figure 3, panel B). Total thiol variation was not related with glycerol doses (Figure 3, panel C). TEAC and MDA did not show changes in respect to the control group (Figure 3, panels D,E).

**Figure 4:**
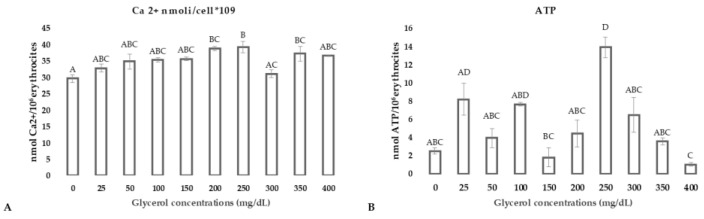
Intracellular Ca^2+^ (**panel A**) and ATP concentration (**panel B**) in RBCs after incubation in media supplemented with glycerol in concentrations ranging from 0 to 400 mg/dL. Uppercase letters indicate significant differences between groups: Ca^2+^ *p* < 0.05 ATP *p* < 0.05; (one-way ANOVA–Tukey post hoc test).

Although in our system we highlighted osmotic and oxidative stress, the intracellular Ca^2+^ ions (Figure 4, panel A) concentration did not change after incubation in media supplemented with glycerol when compared to the control group. Moreover, ATP concentration showed a high variability (Figure 4, panel B) and differences among groups could not be related to glycerol concentrations (Table 3).

As reported in Table 3, the intracellular glycerol concentrations were positively correlated with media osmolality, hemolysis (*p* < 0.01), SOD activity, and MDA concentration (*p* < 0.05). In turn, osmolality and hemolysis were positively correlated with intracellular ROS production (*p* < 0.05), and SOD had a positive correlation with MDA (*p* < 0.05).

**Figure 5:**
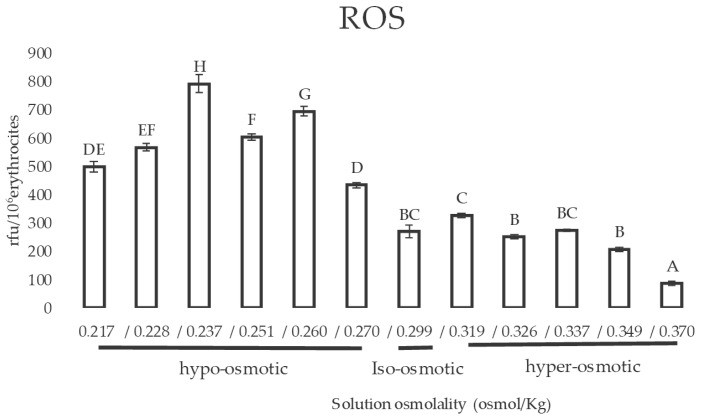
ROS production in sheep RBCs after incubation with 5 different hyper-osmotic solutions and 6 different hypo-osmotic solutions. Uppercase letters indicate significant differences between groups: *p* < 0.05 (one-way ANOVA–Tukey post hoc test).

The effect of cell shrinkage or volume expansion on ROS production has been also studied. As reported in Figure 5, ROS production in RBCs did not change after incubation in hyper-osmotic solutions, showing a significant decrease only at the highest osmolality values. On the other hand, when RBCs were exposed to hypo-osmotic solutions, ROS production increased significantly in all the osmolality values tested.

## 4. Discussion

The EU legislation approved glycerol as an animal feed additive with no restrictions on animal species or quantity that may be fed [45]. However, as recently reported, the increase in plasma glycerol circulating concentrations following its administration may pose negative effects on animal welfare such as increasing plasma osmolality and consequent alterations of RBCs indices [16].

After glycerol oral administration, its concentrations indeed increase sharply in the bloodstream [15,16]. Glycerol is lipid soluble and passes the RBC membrane following a concentration gradient [19]. In sheep, glycerol is transported in RBCs only via simple diffusion [13], and hence it has a lower permeability coefficient compared to humans [14] where it also enters by facilitated diffusion through aquaporins [13]. In this study, we showed that intracellular glycerol concentrations in sheep RBCs increased significantly compared to the control group after incubation in media supplemented with concentrations higher than 200 mg/dL. Above this concentration, hemolysis also increased significantly compared to the control group and a highly positive correlation between hemolysis and intracellular glycerol concentrations was observed. Previous studies reported that glycerol is able to induce hemolysis of mammalian RBCs, likely consequent to the induces osmotic stress [22,23]. This effect is probably correlated to the entry of glycerol into RBCs, which can cause water drawing [45]. Before glycerol and water diffuse into the cells and cause cellular swelling, an osmotically driven water efflux and a concomitant decrease in cell volume occur [46]. In this process, it is necessary to maintain the cell volume within the osmotic tolerance limits, defined as the extent of volume excursions the cell can withstand before irreversible damage occurs [47]. Glycerol is able to induce a severe osmotic stress to the cells [48] and, according to our findings, concentrations higher from 200 mg/mL are above the osmotic tolerance limits of sheep RBCs. In the sheep, these blood concentrations are obtained when administering 170–220 mL of glycerol per dose (22.5–27.4% of the dry matter intake in Sarda adult ewes). Based on our previous findings, lower doses should be preferred for flushing dairy ewes (70 mL, 12.9% of the dry matter intake in Sarda adult ewes), as they proved to be effective at metabolic level without causing alterations in RBC indexes and possibly their functionality [16].

In the present study, each glycerol concentration tested caused a significant increase in the production of intracellular ROS, which was also positively correlated with hemolysis. In other studies, incubation of RBCs with glycerol was not associated with an increase in RBCs’ ROS production [49]. The concentration used in the above-mentioned study (3.5 mg/dL) [49] was, however, significantly lower than the ones tested in the present one. The increase in RBCs’ ROS production found in the present study may thus be linked directly to glycerol entry in the cells, to the alterations in cell volume, or to a combination of both. ROS production may be directly linked to cell swelling as it also increased in hypo-osmotic conditions. This hypothesis is supported by previous studies reporting in various cell types an increase in ROS production following hypotonic exposure [25,26].

However, the increase of SOD activity found at the highest glycerol concentrations, i.e., 350 and 400 mg/dL, suggest that RBCs were able to activate their antioxidant defense systems in response to the glycerol addition in incubation media. Even though total thiols decreased at these concentrations, no changes in MDA intracellular concentrations and total antioxidant capacity were found after incubation in glycerol supplemented media. The increase in MDA concentration is indeed significantly correlated with a decrease in the cell antioxidant capacity [50]. In the present study, RBCs thus proved to be able to cope with the increased ROS production: in fact, MDA did not increase and the antioxidant defenses did not decrease following the glycerol treatment.

Under in vivo conditions, oxidative stress can induce eryptosis through an increase in cellular Ca^2+^ ions [51]. Changes in intracellular Ca^2+^ ions concentrations under stress conditions represent indeed one of the most important trigger mechanisms of metabolic regulation finally underlying death or adaptation of RBCs to the extreme treatments [52]. High intracellular Ca^2+^ ion concentration is responsible for the activation of the phospholipid scramblase [53]. Scramblases destroy the asymmetric distribution of lipids, transferring them from one layer to another by a concentration gradient without energy consumption [54]. These membrane modifications recall macrophages, thus preventing hemolysis from occurring [55]. Instead, under in vitro conditions, damaged erythrocytes cannot be eliminated and undergo hemolysis [33]. In the present study, incubation of RBCs in glycerol supplemented media did not change intracellular Ca^2+^ ion concentration. In the range of glycerol concentrations tested, the RBC systems that control Ca^2+^ ions concentrations thus proved to be able to maintain a low Ca^2+^ level. Erytroptosis can also result from ATP depletion [56]. ATP values found in sheep RBCs in the present study confirm that functional metabolic activity was preserved at all concentrations tested. Therefore, we may speculate that observed hemolysis is caused by osmotic stress, rather than by changes in intracellular Ca^2+^ ions and ATP concentrations.

## 5. Conclusions

In conclusion, results found in the present study showed that the exposition of sheep RBCs to glycerol concentration above 200 mg/dL can cause hemolysis under in vitro conditions. Hemolysis is likely caused by the severe osmotic stress induced by glycerol, rather than by oxidative stress, since RBCs proved to be able to cope with the increased ROS production. The reported increase in ROS production was likely linked to cell swelling consequent to glycerol entry in the cells, even if we cannot rule out a possible direct effect of glycerol. In addition, no alterations in intracellular Ca^2+^ ions and ATP concentrations were found after incubation at the range of glycerol concentrations tested. These results thus suggest that, in sheep, to avoid RBCs’ osmotic stress, glycerol-based nutritional treatments should be designed to reach circulating concentrations not exceeding the limit of 200 mg/dL. This conclusion is supported by a previous in vivo study reporting that when this threshold level is exceeded, RBC shape alterations are observed [16].

## Figures and Tables

**Table 1:** Osmolality values of 5 different hyper-osmotic solutions and 6 different hypo-osmotic solutions. Hyperosmotic solutions were prepared by adding starch to a PBS solution. Hypo-osmotic solutions were prepared adding purified water to a PBS solution.

	Osmol/Kg	Starch Concentrations mg/mL
Hyper-osmotic solutions	0.370	3.50
	0.349	2.91
	0.337	2.23
	0.326	1.69
	0.319	1.44
Iso-osmotic solution	0.299	0.00
Hypo-osmotic solutions	0.270	0.00
	0.260	0.00
	0.251	0.00
	0.237	0.00
	0.280	0.00
	0.217	0.00

**Table 2:** Osmolality values of media supplemented with increasing glycerol concentrations. Uppercase letters indicate differences between groups (*p* < 0.01—one-way ANOVA-Tukey post hoc test).

[Glycerol (mg/dL)]	Osmolality (Osm/Kg) ± S.E.	
0	0.299 ± 0.002	^A^
25	0.312 ± 0.001	^B^
50	0.319 ± 0.001	^BC^
100	0.324 ± 0.002	^CD^
150	0.332 ± 0.002	^DE^
200	0.337 ± 0.001	^EF^
250	0.344 ± 0.002	^FG^
300	0.351 ± 0.002	^GH^
350	0.360 ± 0.001	^HI^
400	0.367 ± 0.002	^I^

**Table 3:** Pearson correlation coefficients between intracellular glycerol, oxidative stress markers, and hemolisys. *p* values are shown within parentheses.

	Glycerol	Osmolality	Haemolysis	ROS	MDA	Ca^2+^ Ions	SOD	Total Thiols	ATP
Osmolality	0.836								
	(0.003)								
Haemolysis	0.915	0.933							
	(<0.001)	(<0.001)							
ROS	0.565	0.853	0.669						
	(0.088)	(0.002)	(0.034)						
MDA	0.631	0.582	0.621	0.382					
	(0.05)	(0.078)	(0.055)	(0.276)					
Ca ^2+^	0.425	0.537	0.393	0.662	0.420				
	(0.221)	(0.11)	(0.262)	(0.037)	(0.227)				
SOD	0.701	0.514	0.573	0.468	0.669	0.279			
	(0.024)	(0.129)	(0.083)	(0.173)	(0.034)	(0.435)			
Total Thiols	−0.282	−0.424	−0.511	−0.455	−0.012	−0.022	−0.083		
	(0.43)	(0.223)	(0.131)	(0.186)	(0.973)	(0.953)	(0.819)		
ATP	−0.274	−0.059	−0.117	0.059	−0.208	0.234	−0.471	−0.108	
	(0.444)	(0.871)	(0.748)	(0.872)	(0.565)	(0.515)	(0.169)	(0.767)	
TEAC	0.248	−0.189	−0.07	−0.327	0.119	0.074	0.357	0.604	−0.049
	(0.489)	(0.6)	(0.847)	(0.356)	(0.742)	(0.839)	(0.311)	(0.064)	(0.893)

## Data Availability

The dataset is available upon request.
